# Establishment and validation of a 3-month prediction model for poor functional outcomes in patients with acute cardiogenic cerebral embolism related to non-valvular atrial fibrillation

**DOI:** 10.3389/fneur.2024.1392568

**Published:** 2024-05-22

**Authors:** Lan Hu, Zhenguo Qiao, Mengshi Xu, Jie Feng, Qingting Shan, Xihua Sheng, Guoli Xu, Yuan Xu, Wenze Hu, Guojun Wang, Xuehong Jin

**Affiliations:** ^1^Department of Neurology, Suzhou Ninth Hospital Affiliated to Soochow University, Suzhou, Jiangsu, China; ^2^Department of Gastroenterology, Suzhou Ninth Hospital Affiliated to Soochow University, Suzhou, Jiangsu, China; ^3^Department of Nursing, Ezhou Polytechnic, Ezhou, Hubei, China; ^4^Department of Neurology, Changshu Hospital Affiliated to Soochow University, Changshu No.1 People’s Hospital, Suzhou, Jiangsu, China; ^5^Department of Neurology, The Affiliated Suzhou Hospital of Nanjing Medical University, Suzhou, Jiangsu, China

**Keywords:** ischemic stroke, cardiogenic cerebral embolism, predictor, outcome, prediction model

## Abstract

**Objectives:**

Cardiogenic cerebral embolism (CCE) poses a significant health risk; however, there is a dearth of published prognostic prediction models addressing this issue. Our objective is to establish prognostic prediction models (PM) for predicting poor functional outcomes at 3 months in patients with acute CCE associated with non-valvular atrial fibrillation (NVAF) and perform both internal and external validations.

**Methods:**

We included a total of 730 CCE patients in the development cohort. The external regional validation cohort comprised 118 patients, while the external time-sequential validation cohort included 63 patients. Multiple imputation by chained equations (MICE) was utilized to address missing values and the least absolute shrink and selection operator (LASSO) regression was implemented through the glmnet package, to screen variables.

**Results:**

The 3-month prediction model for poor functional outcomes, denoted as N-ABCD2, was established using the following variables: NIHSS score at admission (N), Age (A), Brain natriuretic peptide (BNP), C-reactive protein (CRP), D-dimer polymers (D), and discharge with antithrombotic medication (D). The model’s Akaike information criterion (AIC) was 637.98, and the area under Curve (AUC) for the development cohort, external regional, and time-sequential cohorts were 0.878 (95% *CI*, 0.854–0.902), 0.918 (95% *CI*, 0.857–0.979), and 0.839 (95% *CI*, 0.744–0.934), respectively.

**Conclusion:**

The N-ABCD2 model can accurately predict poor outcomes at 3 months for CCE patients with NVAF, demonstrating strong prediction abilities. Moreover, the model relies on objective variables that are readily obtainable in clinical practice, enhancing its convenience and applicability in clinical settings.

## Introduction

Cardiogenic cerebral embolism (CCE) accounts for 20 to 30% of all ischemic strokes (1). Furthermore, up to 60% of patients with embolic stroke of undetermined source (ESUS), constituting 30% to 40% of ischemic strokes (IS), were identified as having cardiogenic origin during follow-up (2), making the cardioembolic stroke population extremely large. Additionally, among all subtypes of IS, CCE is suspected to cause the most significant harm, resulting in an approximate 60% disability and a 20% mortality rate (3).

Numerous prediction models and risk-scoring scales have been developed and validated to predict short or long-term functional outcomes after acute stroke. However, most of these models were established based on the general IS population (4–10), without distinguishing between stroke subtypes, potentially affecting the accuracy of predictions. Meanwhile, as research progresses on cardiogenic cerebral embolism, new biomarkers have been identified as risk factors affecting the prognosis of CCE patients (11, 12). Regrettably, only a few of these new biomarkers have been incorporated into existing stroke prognostic models. It is worth mentioning that whether receiving anticoagulant therapy also has a certain impact on prognosis, but so far, no relevant prognostic model studies have included this variable.

Therefore, there is an urgent need to establish an up-to-date prognostic prediction model for cardiogenic stroke.

## Methods

### Patients

**Figure 1:**
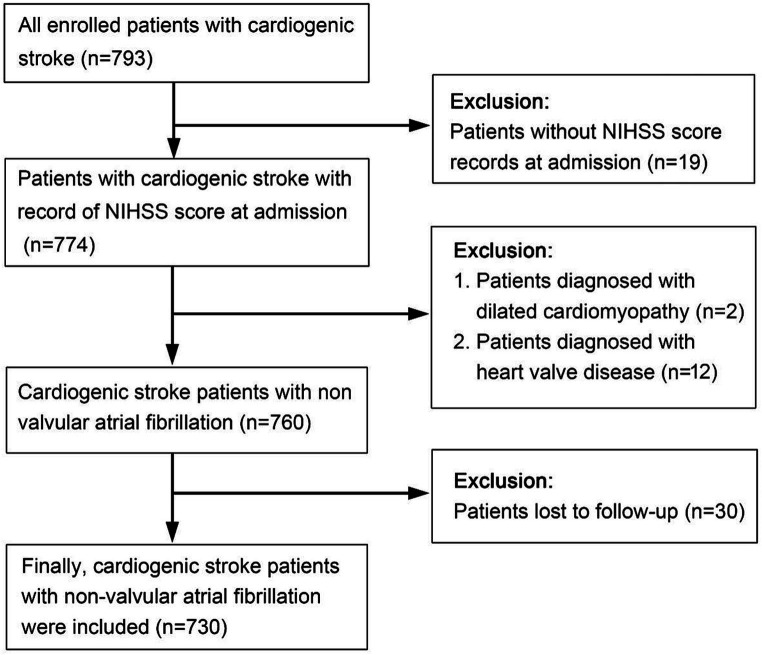
The flowchart of recruitment of the development cohort.

The development cohort consisted of 793 patients with acute CCE who were hospitalized in the Department of Neurology, Suzhou Ninth Hospital affiliated with Soochow University. These patients were retrospectively identified from January 2016 to December 2020. Thirty patients with missing outcome variables, 19 patients without a National Institute of Health stroke scale score (NIHSS) at admission,12 patients with valvular heart disease, and 2 patients with dilated cardiomyopathy were excluded. Finally, 730 patients with acute CCE related to nonvalvular atrial fibrillation (NVAF) were included in the development cohort (Figure 1).

The external regional validation cohort consisted of 118 patients with acute CCE admitted to the Department of Neurology, Changshu No.1 People’s Hospital, from January 2019 to December 2020. The external time-sequential validation cohort comprised 63 CCE patients with NVAF who were hospitalized in the Department of Neurology at Suzhou Ninth Hospital affiliated with Soochow University and were retrospectively identified from January 2021 to July 2021.

Inclusion criteria: (1) Age > 18 years; (2) New infarction confirmed by head computed tomography (CT) or head magnetic resonance (MR)-diffusion-weighted imaging (DWI) within 7 days of onset; (3) History of atrial fibrillation (AF), consistent with acute CCE diagnostic criteria (13); and (4) Provided informed consent.

Exclusion criteria: (1) Patients unable to undergo head CT or MR examination; (2) Those with laboratory and imaging findings meeting the diagnostic criteria for other subtypes of IS; (3) Severe heart valve disease, including rheumatic mitral stenosis, mechanical or biological valve replacement, mitral valve repair, and dilated cardiomyopathy; (4) Patients without NIHSS scores or with missing outcome variables; (5) Participation in other interventional clinical studies within 3 months prior to the date of informed consent or ongoing participating in other interventional clinical research studies; and (6) Patients unwilling to sign the informed consent form.

### Variables selection

The selection of variables was based on indicators related to stroke prognosis as mentioned in previous research and literature, which can be obtained from the electronic medical record system. Variables with a missing proportion exceeding 50% were excluded from candidate predictors. Selected variables included age, sex, history of hypertension or diabetes mellitus, previous stroke or coronary heart disease, peripheral vascular disease, neurological symptoms, initiative blood pressure, ventricular rate, B-type natriuretic peptide (BNP), d-dimer polymers (D-Dimer), C-reactive protein (CRP), serum creatinine (Scr), cardiac troponin I (cTn-I), low-density lipoprotein (LDL), triglycerides, total cholesterol (TC), high-density lipoprotein cholesterol (HDL), left atrial diameter (LAD), left ventricular end systolic diameter (LVDs), left ventricular end diastolic diameter (LVDd), interventricular septal thickness (IVST), left ventricular ejection fraction (LVEF), cranial CT, cranial DWI, chest CT (used to measure left ventricular volume), and three antithrombotic strategies at discharge: no drugs (no oral antithrombotic drugs), oral anticoagulation drugs (oral antiplatelet drugs). Additionally, severity and risk indicators included NIHSS score at admission, CHADS2 score, CHA2DS2-VASc score, and HAS-BLED score.

### Statistical analysis

The event-per-variable approach was employed to assess data sufficiency. All available data from electronic medical record systems were utilized to maximize the statistical power and generalizability of the results. Patient characteristics were summarized as *M* (IQR) or Mean ± SD for continuous variables and as counts and percentages for categorical variables. Multiple imputation by chained equations (MICE) based on R software was used to supplement missing values of baseline variables and parameters (Supplementary Tables 1–3). The distribution of all candidate variables after multiple imputations was comparable to that before imputation. Data cleaning and abnormal value processing were conducted in accordance with the reference ranges provided by each hospital’s testing center.

Binary logistic regression was performed to explore the factors affecting the outcome (mRS > 2). Prior to the regression analysis, all features were standardized to ensure comparability and to mitigate the scale sensitivity inherent in LASSO regression. The least absolute shrinkage and selection operator (LASSO) regression, implemented using the glmnet package, was then employed to screen variables effectively. This approach involved a cross-validation procedure to identify the optimal regularization parameter (λ), thereby balancing the bias-variance tradeoff and enhancing the model’s predictive accuracy.

The final prediction model was derived by refitting the selected variables into a multifactor logistic regression model. Model performance was assessed through measures of discrimination and calibration. Internal validation was performed using a bootstrap procedure (100 resamples) to account for optimism. Discrimination was calculated using the area under Curve (AUC), and calibration accuracy was evaluated using the prognostic index (PI) value calculated by the model.

Statistical analyses were performed using R version 4.0.2, along with packages MICE, rms, and glmnet. The final model was presented in the form of nomographs and a web calculator was developed based on R shiny to facilitate clinical application.

## Results

### Baseline characteristics

The flow diagram illustrating patient selection is shown in Figure 1, and a summary of patient characteristics is presented in Table 1. Within the development cohort, 429 (58.8%) cases, 40 (33.9%) cases in the regional validation cohort, and 23 (36.5%) cases in the time-sequential validation cohort experienced poor functional outcomes (mRS > 2 points) at 3 months. Notably, the proportion of patients with poor functional outcomes in the development cohort was higher than that of the other two cohorts. Correspondingly, there were significant differences in the proportion of patients receiving anticoagulant treatment at discharge among the three cohorts. Specifically, the proportions were as follows: development cohort 16.3% (119/730), regional cohort 14.4% (8/118), and time-sequential validation cohort 46% (29/63) (*p* < 0.001). The proportion of patients receiving anticoagulant therapy in the time-sequential validation cohort was significantly higher than that in the other two cohorts.

**Table 1:** The comparison of clinical data between development cohort and validation cohorts.

Characteristics	Development cohort	External regional cohort	External time sequential cohort	P1-value	P2-value
	*N* = 730	*N* = 118	*N* = 63		
**Gender, *n*(%)**					
Female	367 (50.3)	58 (49.2)	29 (46.0)	0.899	0.607
Male	363 (49.7)	60 (50.8)	34 (54.0)		
Age, mean ± SD	78.8 ± 8.9	77.2 ± 8.5	76.4 ± 8.0	0.076	0.044
**Hypertension, *n*(%)**					
No	170 (23.3)	16 (13.6)	18 (28.6)	0.024	0.344
Yes	560 (76.7)	102 (86.4)	45 (71.4)		
**Diabetes mellitus, *n*(%)**					
No	608 (83.3)	100 (84.7)	55 (87.3)	0.793	0.482
Yes	122 (16.7)	18 (15.3)	8 (12.7)		
**Previous stroke, *n*(%)**					
No	555 (76.0)	89 (75.4)	46 (73.0)	0.979	0.592
Yes	175 (24.0)	29 (24.6)	17 (27.0)		
**CAD, *n*(%)**					
No	570 (78.1)	97 (82.2)	46 (73.0)	0.335	0.354
Yes	160 (21.9)	21 (17.8)	17 (27.0)		
Heart rate, mean ± SD	91.8 ± 20.9	81.5 ± 15.7	83.4 ± 15.2	<0.001	0.002
SBP at admission, mean ± SD	150.2 ± 24.6	148.7 ± 22.1	151.1 ± 23.9	0.534	0.774
DBP at admission, mean ± SD	86.6 ± 15.5	83.8 ± 15.7	85.1 ± 13.0	0.062	0.455
D-dimer, median (P25, P75)	0.8 (0.4, 1.8)	1.11 (0.72, 1.81)	0.25 (0.09, 0.75)	<0.001	<0.001
cTn-I, median (P25, P75)	0.02 (0.01, 0.04)	0.01 (0.01, 0.02)	0.01 (0.00, 0.04)	0.171	0.223
Scr, median (P25, P75)	70.0 (58, 90)	77.5 (62, 88)	67 (57, 85)	0.070	0.839
CRP, median (P25, P75)	4.4 (1.6, 12.9)	1.9 (0.5, 13.0)	3.4 (2.2, 6.9)	<0.001	0.317
BNP, median (P25, P75)	280.3 (161.8, 481.3)	260.5 (155.2, 419.5)	243.0 (150.9, 369.5)	0.394	0.078
LAD, mean ± SD	44.7 ± 6.5	44.8 ± 6.9	44.5 ± 6.9	0.904	0.780
LVDd, mean ± SD	50.5 ± 6.9	45.6 ± 7.0	50.0 ± 6.0	<0.001	0.589
LVDs, median (P25, P75)	34 (31, 39)	29 (26, 32)	33 (30, 36)	<0.001	0.091
IVST, median (P25, P75)	8 (8, 9)	10 (9, 11)	8 (8, 9)	<0.001	0.066
LVEF, mean ± SD	54.1 ± 15.7	63.1 ± 7.8	60.3 ± 7.0	<0.001	0.002
LAV, median (P25, P75)	135.2 (106.1, 167.3)	N/A	153.0 (122.7, 180.7)	N/A	0.006
**NIHSS score group**					
<8 points	433 (59.3)	81(68.6)	43 (68.3)	0.071	0.379
8–15 points	182 (25.0)	10 (8.5)	12 (19.0)		
>15 points	115 (15.7)	27(22.9)	8 (12.7)		
CHADS2, mean ± SD	3.9 ± 0.9	2.7 ± 1.8	3.7 ± 0.8	<0.001	0.088
HAS-BLED, mean ± SD	3.1 ± 0.7	3.0 ± 0.5	3.6 ± 1.0	0.059	<0.001
CHA2DS2-VASc, mean ± SD	5.5 ± 1.2	4.1 ± 1.9	4.7 ± 1.5	<0.001	<0.001
**Discharge medication, *n*(%)**					
None	267 (36.6)	31 (26.3)	9 (14.3)	0.041	<0.001
Antiplatelet drugs	344 (47.1)	70 (59.3)	25 (39.7)		
Anticoagulant drugs	119 (16.3)	17 (14.4)	29 (46.0)		
mRS				<0.001	0.001
≤2 points	301 (41.2)	78 (66.1)	40 (63.5)		
>2 points	429 (58.8)	40 (33.9)	23 (36.5)		

SBP, Systolic blood pressure; DBP, Diastolic blood pressure; Scr, Serum creatinine; BNP, Brain natriuretic peptide; CRP, C-reactive protein; SD, Standard deviation; cTn-I, Cardiac troponin I; LAD, Left atrial diameter; LAV, Left atrial volume; LVDs, Left ventricular end systolic diameter; LVDd, Left ventricular end diastolic diameter; IVST, Interventricular septal thickness; LVEF, Left ventricular ejection fraction; NIHSS, National Institute of Health stroke scale; CHADS2, congestive heart failure, hypertension, age, diabetes, prior stroke are each assigned 1 point; CHA2DS2-VASc, congestive heart failure, hypertension, diabetes, 65–74 years of age, female sex, and vascular disease are each assigned 1 point, and prior stroke or transient ischemic attack and being 75 years of age or older are assigned 2 points; HAS-BLED, hypertension, abnormal renal/liver function, stroke, bleeding history or predisposition to bleeding, labile international normalized ratio, elderly, and drugs/alcohol concomitantly are each assigned 1 point; mRS, Modified Rankin Scale; N/A, Not Applicable.

Regarding missing data, there were 49 cases lacking D-dimer values in the development cohort but not in the regional and time-sequential cohorts. The deficiency of BNP data was higher in the development cohort (11%) compared to the regional (4.8%) and time-sequential cohorts (1.7%). Regarding the absence data of cTn-I data, the rates of missing values differed among the cohorts: development cohort (15.5%), regional validation cohort (4.8%), and time-sequential cohort (16.9%). Among all clinical data, the most serious deficiency was found in cardiac ultrasound indicators (LAD, LVDs, LVDd, IVST, LVEF) (46.7%). After imputing missing values, all eligible patients were included for model development or validation. Eight variables (D-dimer, cTn-I, Scr, CRP, BNP, LVDs, IVST, LAV) did not conform to a linear distribution and were logarithmically processed.

A total of 29 variables [gender, age, hypertension, diabetes mellitus, previous stroke history, compliance coronary artery disease (CAD), heart rate at admission, systolic blood pressure (SBP) at admission, diastolic blood pressure (DBP) at admission, log D-dimer, log cTn-I, log serum creatinine, log CRP, log BNP, LVDd, log LVDs, log IVST, log LAV, LAD, LVEF, NIHSS score group (<8 points, 8–15 points, >15 points), CHADS2 score, HAS-BLED score, CHA2DS2-VASC score, and discharge medication (no antithrombotic drugs, antiplatelet drugs, and oral anticoagulant drugs)] were used to construct the LASSO logistic regression model (Supplementary Figure 1).

**Figure 2:**
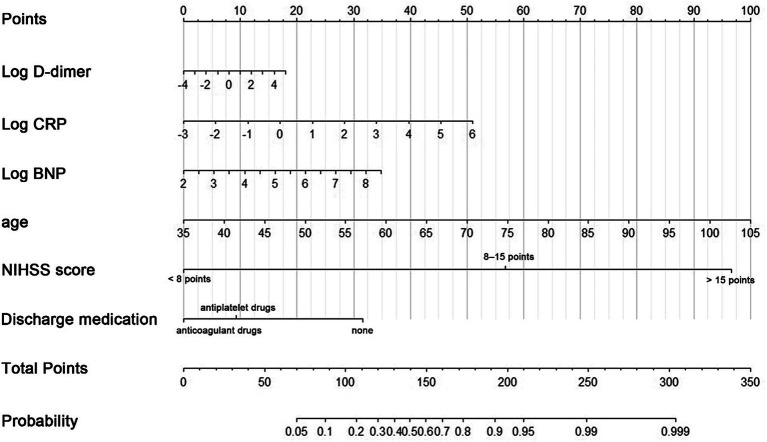
A nomogram predicting the probability of poor functional outcomes (mRS > 2) in CCE patients related to NVAF. Draw an upward vertical line to the “Points” bar to calculate points. Based on the sum, draw a downward vertical line from the “Total Points” line to calculate.

Based on the results of variable screening using LASSO and clinical practice, six predictors, including age, log D-dimer, log CRP, log BNP, NIHSS score group (< 8 points, 8–15 points, > 15 points), and discharge medication (no antithrombotic drugs, antiplatelet drugs, or oral anticoagulant drugs) were selected to construct the N-ABCD2 model (N-NIHSS at admission; A-Age; B-BNP; C-CRP; D-D-dimer; D-Discharge medication; Table 2; Figure 2). The Akaike information criterion (AIC) of the N-ABCD2 model was 637.98. Additionally, we established a free web page for model calculation.^1^ By entering relevant variable information on the web page, users can quickly obtain the risk prediction value for the case.

$$\begin{array}{l} \mathrm{Prognostic index}\;\left(\mathrm{PI}\right)=-6.06216+0.06013*\mathrm{Age}+0.08389*\\ \log D-d\mathrm{imer}+0.23877*\log\mathrm{CRP}+\\ 0.22598*\log\mathrm{BNP}+2.38593\;\\ \left(\mathrm{If NIHSS score was}\;8-15\;\mathrm{points}\right)+\\ 4.06893\;\left(\mathrm{If NIHSS score}>15\;\mathrm{points}\right)-\\ 0.93617\;\left(\begin{array}{l} \mathrm{If discharge medication}\\ \mathrm{was antiplatelet drugs} \end{array}\right)-\\ 1.32821\;\left(\begin{array}{l} \mathrm{If discharge medication}\\ \mathrm{was anticoagulant drugs} \end{array}\right) \end{array}$$

$$\mathrm{Event probability}=\frac{e^{PI}}{1+e^{PI}}$$

**Table 2:** Fitting results of the N-ABCD2 model.

Intercept and predictors	*β*	*OR*	95%*CI*	*P*-value
Intercept	−6.062			
Age	0.060	1.062	1.036–1.090	<0.001
Log D-dimer	0.084	1.088	0.907–1.303	0.363
LogCRP	0.239	1.270	1.102–1.467	0.001
LogBNP	0.226	1.254	0.980–1.608	0.073
NIHSS score				
<8 points	1.00			
8–15 points	2.386	10.869	6.479–19.095	<0.001
>15 points	4.069	58.494	1.725–366.942	<0.001
Discharge medication				
None	1.00			
Antiplatelet drugs	−0.936	0.392	0.243–0.625	<0.001
Anticoagulant drugs	−1.328	0.264	0.142–0.486	<0.001

BNP, Brain natriuretic peptide; CRP, C-reactive protein; OR, Odds ratio; CI, confidence interval.

In this formula, NIHSS score and discharge medication were considered dummy variables, with a value of 1 indicating satisfaction with the condition and 0 indicating no satisfaction.

### Evaluation of N-ABCD2 model

**Figure 3:**
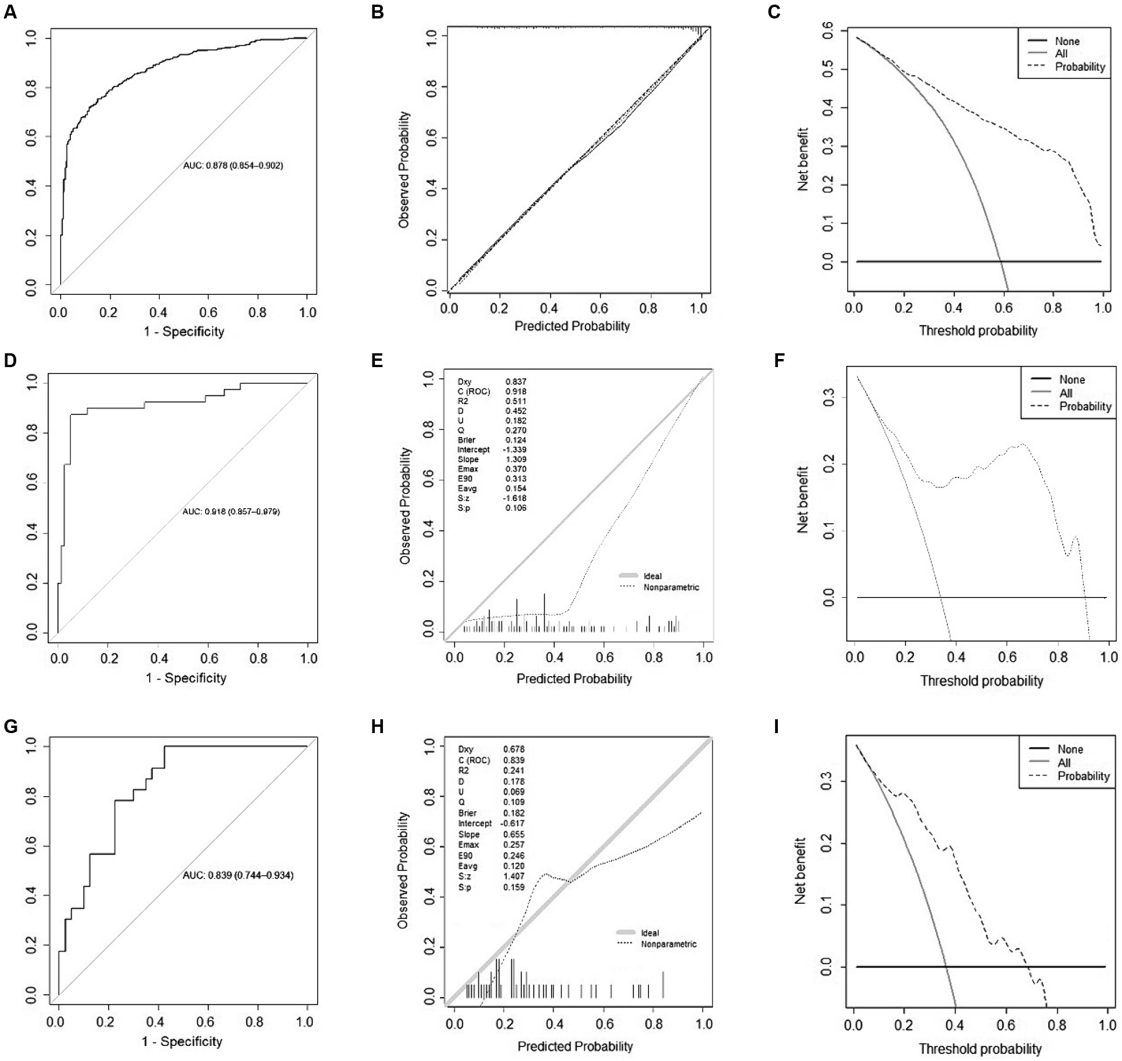
Model evaluation of development and validation cohort. **(A)** Receiver Operating Characteristic (ROC) Curve for the development cohort; **(B)** Calibration curve for the development cohort; **(C)** Decision curve analysis for the development cohort; **(D)** ROC Curve for the external regional validation cohort; **(E)** Calibration curve for the external regional validation cohort; **(F)** Decision curve analysis for the external regional validation cohort; **(G)** ROC Curve for the time sequential validation cohort; **(H)** Calibration curve for the time sequential validation cohort; **(I)** Decision curve analysis for the time sequential validation cohort.

The receiver operating characteristic curve (ROC curve; Figure 3A) and the calibration curve (Figure 3B) for the development cohort were constructed. The AUC for the development cohort was 0.878 (95% *CI*, 0.854–0.902), indicating high discrimination. The *p*-value of the Hosmer-Lemeshow (H-L) test was 0.714, and the Brier score was 0.139, suggesting that the model did not exhibit overfitting and had good extrapolation performance. Decision curve analysis (DCA) was employed to assess the net benefit, as shown in Figure 3C. The threshold probability range for patient net benefit essentially covered 0–1.0, signifying that the N-ABCD2 model provided high net benefit.

### Validation of N-ABCD2 model

In the external regional validation cohort, the AUC of the model remained high at 0.918 (95% *CI*, 0.857–0.979), with discrimination slightly higher than that of the development cohort (Figure 3D). The *p*-value of the H-L test was 0.106, indicating no statistical significance between predicted and actual observations. The Brier score was 0.124 (Figure 3E), suggesting that the predicted probability from the N-ABCD2 model was somewhat higher than the actual probability for this validation set, indicating a tendency to overestimate the risk of poor function when using this model.

Compared to the development cohort, the DCA curve of the regional validation cohort is presented in Figure 3F. The situation was essentially the same as that of the time-sequential validation cohort. After the threshold probability exceeded 0.7, patients failed to obtain net benefit from a model evaluation, and the degree of net benefit was significantly lower than that of the development cohort.

The ROC curve and a calibration curve of the time-sequential validation cohort are shown in Figure 3G. In the external time-sequential validation cohort, the AUC of the model was 0.839 (0.744–0.934), with discrimination not significantly lower than that of the development cohort. The *p*-value of the H-L test was 0.159, indicating no statistically significant difference between predicted and actual observations. The Brier score was 0.182. However, the prediction probability distribution of the validation set was imbalanced, with the model slightly inaccurate when the prediction probability was lower than 0.1 or higher than 0.8 (Figure 3H). Compared to the development cohort, the DCA curve for the period validation cohort is shown in Figure 3I. Beyond a probability threshold of 0.7, did not benefit from the model evaluation, and the degree of net benefit was also significantly lower than that observed in the development cohort. Nevertheless, when considering the external time-sequential cohort as a whole, the N-ABCD2 model continued to exhibit a high degree of discrimination and calibration. Patients could derive a net benefit from the model evaluation within the probability range of 0.1–0.7.

## Discussion

Different subtypes of IS have distinct pathogenesis and outcomes, necessitating more refined prediction models for stroke prognosis to accurately predict outcomes in different IS populations.

Our study significantly differs from previous research on stroke prognosis. Firstly, our study focuses on acute CCE patients related to NVAF rather than a general ischemic stroke population. The N-ABCD2 model includes six variables: NIHSS score at admission, age, BNP, CRP, D-Dimer polymers, and antithrombotic selection at discharge. Of these, only age is a traditional risk factor, with the others being new risk factors mentioned in the literature (14–17). Some of these new risk factors have been reported in previous models, emphasizing their importance (9, 11, 18–21). Beatty’s study (22) compared traditional and new risk factors as predictors of cardiovascular events in patients with stable coronary artery disease (CAD). The top 4 predictors were N-terminal proBNP, high-sensitivity cardiac troponin T (hs-cTnT), urinary albumin to creatinine ratio, and smoking, outperformed traditional risk factors (age, sex, body mass index, hypertension, dyslipidemia, and diabetes) in predicting 5-year risk of secondary events in patients with stable coronary heart disease (CHD). In comparison to the variables in the Framingham secondary events model, the Heart and Soul risk model yielded a net reclassification improvement of 0.47 (95% *CI*, 0.25–0.73) in the derivation cohort and 0.18 (95% *CI*, 0.01–0.40) in the validation cohort. Furthermore, as reported in a separate study (23), there exists a clear gradient relationship between the number of elevated novel biomarkers and the risk of major disability, mortality, and vascular events. The incorporation of a combination of multiple biomarkers substantially improved the risk stratification for adverse outcomes in IS patients, reaffirming the importance and necessity of including various novel biochemical markers in prognostic prediction models.

GPS-GF score (24) was the first predictive model for the 30-day death of patients with AF related CCE in China, which includes five predictors: gender, Glasgow coma scale (GCS) score, complicated pneumonia, midline shift of head imaging examination (CT or MR) of 10 mm, and blood glucose level. Different from the N-ABCD2 model, GPS-GF score included patients with AF related ischemic stroke, including valvular AF and NVAF (accounted for 65%), while in the N-ABCD2 model, all patients related with NVAF. Interestingly, the variables of the two models were completely different, which may be related to the composition of study population, sample size, the predicted outcome variables are different.

The N-ABCD2 model is based on age and NIHSS score at admission, supplemented by four biochemical indicators, BNP, CRP, and D-Dimer from distinct pathways to predict outcomes. Notably, the N-ABCD2 model also incorporates a unique variable: antithrombotic strategy at discharge, a feature rarely reported in previous stroke prognosis models. It is widely acknowledged that timely and standardized anticoagulant treatment is a vital factor in the prognosis of CCE patients. In clinical practice, many factors such as efficacy, cost, availability of drugs, patients’ compliance with treatment, bleeding risk, whether anticoagulants exist reversal agents and comorbidity should be considered when selecting anticoagulant therapy, and patients’ options should also be considered. In this study, the development cohort consisted of patients with NVAF-related CCE who were hospitalized between January 2016 and December 2020, and the data showed a lower proportion (16.3%) of the development cohort received anticoagulant therapy at discharge, the rate of 16.3% only represented the anticoagulation situation at the time of discharge, some patients with delayed anticoagulation (due to acute hemorrhage transformation or complicated bleeding events) or subsequent adjustment from antiplatelet to anticoagulation strategy were not included. The N-ABCD2 model had showcased good ability to adapt to the current landscape while exhibiting strong predictive performance, discrimination, and calibration. Importantly, a good prediction model should be easily applicable. The variables in the N-ABCD2 model are typically collected as part of routine clinical practice, resulting in minimal associated costs. Theoretically, this model holds great potential for widespread use. Through the N-ABCD2 model, medical professionals, patients, or their families can accurately calculate the probability of a poor prognosis within 3 months using our online computing tools, facilitating more informed medical decisions in the future.

There are still some limitations in this study: First of all, IS is a dynamic condition, and blood markers change accordingly over time, this study included patients with cardiogenic stroke whose onset occurred within 1 week, potentially introducing variability due to variations in blood-related indicators collected at different time points. Secondly, our study primarily focused on biochemical markers related to inflammation, coagulation, fibrinolysis, and heart, and kidney function. Future research could delve deeper into markers affecting the integrity of the blood–brain barrier, such as matrix metalloproteinase-9 (MMP-9), and their impact on prognosis. Thirdly, the proportion of anticoagulation in the development cohort and the regional validation cohort in this study were low, which may have a certain impact on the prognosis and our study did not consider the impact of complications on prognosis.

In conclusion, our research is a foundational step toward optimizing stroke prediction models in the future. It underscores the importance of secondary prediction and prevention, particularly in predicting the prognosis of NVAF-related CCE patients through the integration of multiple related biochemical and imaging markers. Future studies should prioritize larger sample sizes and the inclusion of novel predictors, potentially including genomics factors.

The N-ABCD2 model can specifically predict poor outcomes in CCE patients with NVAF at 3 months. The model has strong prediction abilities, discrimination, and calibration. The model’s predictive variables are objective and easily attainable in clinical practice, rendering it a convenient tool for widespread clinical application.

## Data availability statement

The original contributions presented in the study are included in the article/Supplementary material, further inquiries can be directed to the corresponding authors.

## Ethics statement

The studies involving humans were approved by Suzhou Ninth Hospital Affiliated to Soochow University (Suzhou, China) and Changshu No.1 People’s Hospital (Suzhou, China). The studies were conducted in accordance with the local legislation and institutional requirements. The participants provided their written informed consent to participate in this study. Written informed consent was obtained from the individual(s) for the publication of any potentially identifiable images or data included in this article.

## Author contributions

LH: Funding acquisition, Writing – original draft. ZQ: Writing – original draft. MX: Data curation, Formal analysis, Writing – review & editing. JF: Data curation, Formal analysis, Writing – review & editing. QS: Investigation, Methodology, Writing – review & editing. XS: Investigation, Methodology, Writing – review & editing. GX: Software, Validation, Writing – review & editing. YX: Supervision, Visualization, Writing – review & editing. WH: Writing – review & editing. GW: Conceptualization, Writing – review & editing. XJ: Conceptualization, Writing – review & editing.

## Footnotes

^1^ https://prediction1.shinyapps.io/mrsprediction/
