# Supplementary material for: Establishment and validation of a 3-month prediction model for poor functional outcomes in patients with acute cardiogenic cerebral embolism related to non-valvular atrial fibrillation
Source: Front Neurol. 2024 May 22;15:1392568. doi: 10.3389/fneur.2024.1392568 (PMC11150815; doi:10.3389/fneur.2024.1392568)
Supplement: Supplementary file 1 [file Data_Sheet_1.PDF]

Supplementary Material

1 Supplementary Figures and Tables

1.1 Supplementary Figures

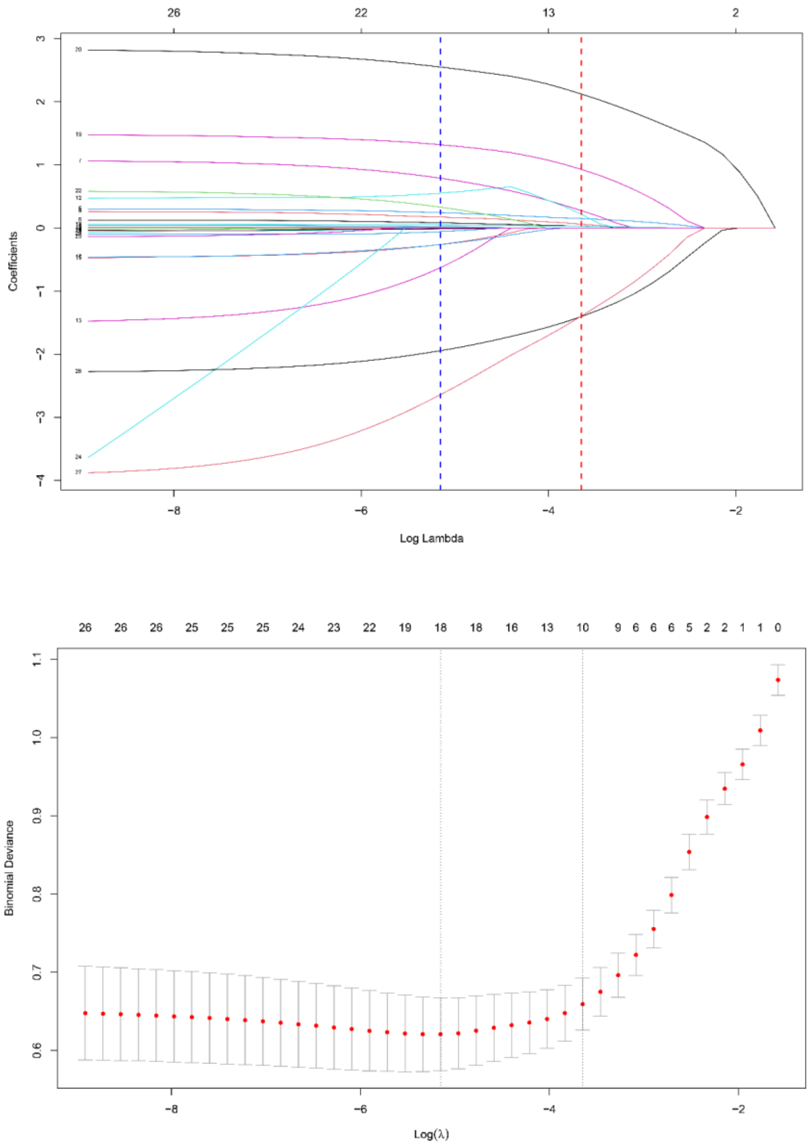

Supplementary Figure 1. Texture feature selection using the least absolute shrinkage and selection operator (LASSO) binary logistic regression model
