# Supplementary material for: Establishment and validation of a 3-month prediction model for poor functional outcomes in patients with acute cardiogenic cerebral embolism related to non-valvular atrial fibrillation
Source: Front Neurol. 2024 May 22;15:1392568. doi: 10.3389/fneur.2024.1392568 (PMC11150815; doi:10.3389/fneur.2024.1392568)
Supplement: Supplementary file 2 [file Data_Sheet_2.PDF]

1.2 Supplementary Tables

Supplementary Table 1. The comparison of the development and external regional validation cohorts before and after multiple imputation

Characteristics	Missing values (%)
D-dimer	49(6.7)
Troponin I	113(15.5)
CRP	21(2.9)
BNP	80(11.0)
LAD	341(46.7)
LVDd	341(46.7)
LVDs	341(46.7)
IVSTD	341(46.7)
LVEF	341(46.7)
LAV	341(46.7)
