# Supplementary material for: Establishment and validation of a 3-month prediction model for poor functional outcomes in patients with acute cardiogenic cerebral embolism related to non-valvular atrial fibrillation
Source: Front Neurol. 2024 May 22;15:1392568. doi: 10.3389/fneur.2024.1392568 (PMC11150815; doi:10.3389/fneur.2024.1392568)
Supplement: Supplementary file 3 [file Data_Sheet_3.PDF]

Supplementary Table 2. The comparison of the development and external regional validation cohorts before and after multiple imputation

Characteristics	Before multiple imputation			After multiple imputation		
	Development cohort N=730	External regional cohort N=118	P-value	Development cohort N=730	External regional cohort N=118	P-value
D2,median[P25,P75]	0.8 [0.4, 1.8]	1.1 [0.7, 1.8]	<0.001	0.8 [0.4, 1.8]	1.11 [0.72, 1.81]	<0.001
Troponin I,median[P25,P75]	0.0 [0.0, 0.0]	0.0 [0.0, 0.0]	<0.001	0.02 [0.01, 0.04]	0.01 [0.01, 0.02]	0.171
CRP,median[P25,P75]	4.5 [1.6, 12.9]	1.9 [0.5, 13.0]	<0.001	4.4 [1.6, 12.9]	1.9 [0.5, 13.0]	<0.001
BNP,median[P25,P75]	283.7 [167.4, 490.1]	256.5 [155.2, 419.5]	0.196	280.3 [161.8, 481.3]	260.5 [155.2, 419.5]	0.394
LAD,mean±SD	44.9±6.4	44.8±6.9	0.829	44.7±6.5	44.8±6.9	0.904
LVDd,mean±SD	49.9±6.5	45.7±7.0	<0.001	50.5±6.9	45.6±7.0	<0.001
LVDs,median[P25,P75]	33.0 [30.0, 38.0]	29.0 [26.0, 32.0]	<0.001	34 [31, 39]	29 [26, 32]	<0.001
IVSTD,median[P25,P75]	8.0 [8.0, 9.0]	10.0 [9.2, 11.0]	<0.001	8 [8, 9]	10 [9, 11]	<0.001
EF,mean±SD	55.3±15.5	63.1±7.8	<0.001	54.1±15.7	63.1±7.8	<0.001
LAV,median[P25,P75]	135.2 [106.1, 167.3]	N/A	N/A	135.2 [106.1, 167.3]	N/A	N/A
