# Supplementary material for: Establishment and validation of a 3-month prediction model for poor functional outcomes in patients with acute cardiogenic cerebral embolism related to non-valvular atrial fibrillation
Source: Front Neurol. 2024 May 22;15:1392568. doi: 10.3389/fneur.2024.1392568 (PMC11150815; doi:10.3389/fneur.2024.1392568)
Supplement: Supplementary file 4 [file Data_Sheet_4.PDF]

Supplementary Table 3. The comparison of the development and external time sequential validation cohorts before and after multiple imputation

Characteristics	Before multiple imputation			After multiple imputation		
	Development cohort N=730	External time sequential cohort N=63	P-value	Development cohort N=730	External time sequential cohort N=63	P-value
D2,median[P25,P75]	0.8 [0.4, 1.8]	0.3 [0.1, 0.8]	<0.001	0.8 [0.4, 1.8]	0.25 [0.09, 0.75]	<0.001
Troponin I,median[P25,P75]	0.0 [0.0, 0.0]	0.0 [0.0, 0.0]	<0.001	0.02 [0.01, 0.04]	0.01 [0.00, 0.04]	0.223
Scr,median[P25,P75]	70.0 [58.0, 90.0]	67.0 [57.5, 85.5]	0.811	70.0 [58, 90]	67 [57, 85]	0.839
CRP,median[P25,P75]	4.5 [1.6, 12.9]	3.4 [2.2, 7.4]	0.347	4.4 [1.6, 12.9]	3.4 [2.2, 6.9]	0.317
BNP,median[P25,P75]	283.7 [167.4, 490.1]	259.0 [156.9, 411.2]	0.519	280.3 [161.8, 481.3]	243.0 [150.9, 369.5]	0.078
LAD,mean±SD	44.9±6.4	44.5±6.9	0.594	44.7±6.5	44.5±6.9	0.780
LVDd,mean±SD	49.9±6.5	50.0±6.0	0.936	50.5±6.9	50.0±6.0	0.589
LVDs,median[P25,P75]	33.0 [30.0, 38.0]	32.0 [30.0, 35.5]	0.33	34 [31, 39]	33 [30, 36]	0.091
IVSTD,median[P25,P75]	8.0 [8.0, 9.0]	8.0 [8.0, 9.0]	0.044	8 [8, 9]	8 [8, 9]	0.066
EF,mean±SD	55.3±15.5	60.3±7.0	0.012	54.1±15.7	60.3±7.0	0.002
LAV,median[P25,P75]	135.2 [106.1, 167.3]	151.7 [120.7, 179.2]	0.052	135.2 [106.1, 167.3]	153.0 [122.7, 180.7]	0.006
